# Shifting gender norms to improve HIV service uptake: Qualitative findings from a large-scale community mobilization intervention in rural South Africa

**DOI:** 10.1371/journal.pone.0260425

**Published:** 2021-12-31

**Authors:** Anna M. Leddy, Ann Gottert, Nicole Haberland, Jennifer Hove, Rebecca L. West, Audrey Pettifor, Sheri A. Lippman, Kathleen Kahn, Rhandzekile Mathebula, Dumisani Rebombo, Xavier Gómez-Olivé, Rhian Twine, Dean Peacock, Julie Pulerwitz

**Affiliations:** 1 Division of Prevention Science, Department of Medicine, University of California San Francisco, San Francisco, CA, United States of America; 2 Population Council/Project SOAR, Washington, DC and New York, NY, United States of America; 3 MRC/Wits Rural Public Health and Health Transitions Research Unit (Agincourt), School of Public Health, Faculty of Health Sciences, University of the Witwatersrand, Johannesburg, South Africa; 4 Department of Epidemiology, University of North Carolina at Chapel Hill, Chapel Hill, NC, United States of America; 5 Sonke Gender Justice, Johannesburg, South Africa; 6 Promundo, Washington, D.C., United States of America; Centre for Sexual Health & HIV/AIDS Research (CeSHHAR)R, ZIMBABWE

## Abstract

**Background:**

Interventions to improve HIV service uptake are increasingly addressing inequitable and restrictive gender norms. Yet comparatively little is known about which gender norms are most salient for HIV testing and treatment and how changing these specific norms translates into HIV service uptake. To explore these questions, we implemented a qualitative study during a community mobilization trial targeting social barriers to HIV service uptake in South Africa.

**Methods:**

We conducted 55 in-depth interviews in 2018, during the final months of a three-year intervention in rural Mpumalanga province. Participants included 25 intervention community members (48% women) and 30 intervention staff/community-opinion-leaders (70% women). Data were analyzed using an inductive-deductive approach.

**Results:**

We identified three avenues for gender norms change which, when coupled with other strategies, were described to support HIV service uptake: (1) Challenging norms around male toughness/avoidance of help-seeking, combined with information on the health and preventive benefits of early antiretroviral therapy (ART), eased men’s fears of a positive diagnosis and facilitated HIV service uptake. (2) Challenging norms about men’s expected control over women, combined with communication and conflict resolution skill-building, encouraged couple support around HIV service uptake. (3) Challenging norms around women being solely responsible for the family’s health, combined with information about sero-discordance and why both members of the couple should be tested, encouraged men to test for HIV rather than relying on their partner’s results. Facility-level barriers such as long wait times continued to prevent some men from accessing care.

**Conclusions:**

Despite continued facility-level barriers, we found that promoting critical reflection around several specific gender norms, coupled with information (e.g., benefits of ART) and skill-building (e.g., communication), were perceived to support men’s and women’s engagement in HIV services. There is a need to identify and tailor programming around specific gender norms that hinder HIV service uptake.

## Introduction

Evidence suggests that efforts to improve engagement in HIV care and treatment should address inequitable and restrictive gender norms [1–4]. These gender norms can condone men’s dominance of household decision-making and their use of violence to exert control over women [5, 6]. They can also include norms around men’s need to demonstrate ‘toughness’ and their role as the primary family provider, and women’s role as the sole caregiver for children [7]. These gender norms that reproduce and support power hierarchies between men and women (i.e., gender inequality) are prevalent in South Africa, and contribute to HIV risk in this setting- where the burden of HIV is the highest globally [8–14]. Recent studies have found that inequitable gender norms also prevent men and women from engaging in HIV care and treatment [1–3]. Qualitative research suggests that men who endorse inequitable gender norms are less likely to access health services including HIV testing, care and treatment because seeking help is viewed as a sign of weakness and is in conflict with the masculine ideal of self-reliance and toughness [2, 4]. Further, men who endorse such gender norms are more likely to perpetrate violence against their female partners [15], which prevents women’s engagement in HIV care and treatment [16, 17]. However, other studies from sub-Saharan Africa contribute more nuanced findings, suggesting that inequitable gender norms are not invariably associated with decreased use of health services among men, and highlight the important role of clinic-level barriers in preventing men’s service uptake [3, 18].

Community mobilization strategies, which seek to bring communities together to spark dialogue and collective action to achieve a shared goal [19], have demonstrated promise in altering inequitable and harmful gender norms, and reducing intimate partner violence (IPV) and HIV risk [20–24]. A growing body of evidence also suggests that community mobilization can improve engagement along the HIV care cascade- or the steps people living with HIV take from HIV testing and diagnosis, linkage to and retention in HIV care, to achieving and maintaining viral suppression [21, 25, 26]. However, our understanding of the effect of such multi-faceted interventions on service uptake, and the process through which this occurs, is limited [27]. A deeper understanding of how community mobilization and other multifaceted community-based approaches shift gender norms among men and women can delineate which gender norms change and how this can lead to HIV service uptake [28, 29]. Identifying the specific norms that need to be addressed to facilitate HIV service uptake, and the pathways through which this can be achieved, is critical to inform targeted intervention efforts.

*Tsima* (“Working together”) [30] was a community mobilization intervention that sought to improve both men’s and women’s engagement in HIV testing, care, and treatment by addressing social barriers that deter service uptake including gender norms. It was implemented over three years in rural Mpumalanga province, South Africa. We implemented a qualitative study during the Tsima community mobilization trial to assess whether and how relevant gender norms were shifting, and to understand whether and how shifting norms were impacting HIV service uptake. Other recent quantitative work by our team in this study area suggests that certain gender norms—including norms around men’s toughness, men’s control over women in relationships and women’s primary role as caretaker in the family—are endorsed by most men and women, with implications for HIV service uptake [3]. Consequently, we explored Tsima staff and participants’ views on whether and how Tsima changed these kinds of norms, and the pathways through which this may have led to HIV service uptake.

## Methods

### Study setting

The Tsima intervention was implemented in the Bushbuckridge subdistrict of rural Mpumalanga province in northeast South Africa from 2015 to 2018. This area includes the Agincourt Health and Socio-Demographic Surveillance System (HDSS) site, which has been run by the Medical Research Council-Wits University Rural Public Health and Health Transitions Research Unit since 1992. The HDSS includes 31 villages, 15 of which participated in the Tsima trial and were randomized to either the intervention (n = 8) or control (n = 7) arm. HIV testing and care are provided to HDSS residents through nine public health clinics located within the study area. Population-based estimates of HIV prevalence in the Agincourt HDSS suggest that 19.4% of adults 15 years and older are living with HIV [31].

### Description of intervention

Tsima was a community mobilization intervention designed to improve HIV testing, care and treatment outcomes among adults aged 18–49 years, as described in detail elsewhere [30]. It specifically sought to address social barriers to engaging in HIV services, such as limited knowledge about treatment as prevention, poor treatment literacy, HIV stigma and fear of disclosure, and inequitable gender norms. The intervention was not designed to provide HIV services, nor make changes to clinics to facilitate HIV service uptake; rather, community members accessed the largely facility-based HIV services available in their communities.

Intervention activities were informed by a community mobilization model grounded in social science theory and validated in the study community [19, 30, 32], and adapted from Sonke Gender Justice’s One Man Can campaign [33], which had previously been implemented and evaluated by the study team in different villages covered by the Agincourt HDSS [24]. Tsima was designed to spark community dialogue around HIV testing, linkage to and retention in care, and gender equity through intervention activities including two-day workshops, door-to-door outreach, digital stories screenings, young women’s groups, community murals and soccer and other events; engaging local leadership; and fostering community cohesion to support people living with HIV [30]. Throughout all workshops and many other intervention activities, Tsima aimed to change gender norms that discourage engagement in care, including norms condoning men’s toughness, men as the primary decision-maker, men’s use of violence and control over women, women’s sole responsibility for caregiving, and to reinforce communication skills to achieve healthy partnerships.

Tsima was led by two managers and a team of 18 community mobilizers, most of whom were from the intervention communities and assigned to work in their own villages whenever feasible. The community mobilizers also identified and trained Community Action Team (CAT) members in each village. CAT members were volunteers from the community who worked closely with community mobilizers to implement intervention activities and mobilization efforts in their communities.

A cluster randomized trial design (R01MH103198) was used to assess the effectiveness of the Tsima intervention in improving HIV testing, linkage, and retention in HIV care among individuals (18–49 years) residing in the eight intervention communities compared to individuals residing in seven control communities in the Agincourt HDSS. Details related to the trial design are described elsewhere [30]. Endline trial data showed markedly higher uptake across the HIV care cascade among men and women, in intervention vs. control communities [34].

### Sampling and recruitment

With additional funding from PEPFAR through Project SOAR (led by the Population Council), we conducted a qualitative study to examine the gender norm change process and how such changes influenced HIV service utilization. Data for this paper comes from qualitative in-depth interviews (IDIs) conducted early to mid-2018, in the final months of the three-year intervention. These IDIs focused specifically on the process of gender norms change and associated changes in HIV service uptake. IDIs for the present study were conducted with 25 community members (48% women; 60% HIV-positive) and 30 key informants (70% women), for a total of 55 interviews. Key informants included community mobilizers (n = 16), CAT members (n = 7), and community opinion leaders (n = 7).

For the community member sample, CAT and mobilization teams helped identify participants aged 18–49 (the target population for Tsima), who resided in intervention communities and had participated in at least one Tsima activity. Participants were purposively sampled to obtain equal representation of males and females and HIV-negative and HIV-positive participants using data collected during the trial.

For the key informant sample, all community mobilizers were invited to participate. CAT members and community leaders (e.g., local government officials, religious leaders, and representatives of community-based organizations) were purposively selected by the study team based on their level of engagement in the community and the intervention.

The study team contacted each participant, described the nature of the qualitative study, and asked them to participate in the interview. If the participant agreed, they arranged a suitable date and place for the interview.

### Data collection

Semi-structured interview guides were developed for community members and key informants and explored gender norms in the local context, Tsima’s effect on gender norms, IPV, HIV testing, HIV status disclosure and treatment uptake. Community members were also asked about their personal experiences with Tsima including the effect Tsima had on their own HIV testing, disclosure, treatment, caregiving, and relationships including IPV. Each of the interview guides were piloted prior to use with 2–3 participants and revised accordingly.

All interviews were conducted in Tsima program offices or a private location of the participant’s choosing. Interviews were conducted by three experienced female qualitative interviewers resident in the study area, in either the local language (Shangaan) or English depending on the participant’s preference. Interviewers received comprehensive training in research ethics and topics relevant to the study, including the content of the intervention, concepts related to gender norms, HIV stigma and HIV testing, care and treatment. Interviews lasted approximately one hour and were audio-recorded, transcribed and translated from Shangaan to English as necessary.

### Data analysis

All interview transcripts were entered into ATLAS.ti [35] qualitative analysis software for coding. Analysis was conducted using an inductive-deductive approach and facilitated by multiple readings of the transcripts and memo writing to highlight emergent themes [36]. An initial coding schema was developed based on *a priori* codes informed by the research questions and literature review. The coding schema was then iteratively revised by adding new codes that reflected additional themes that emerged from the data. The researchers met regularly throughout this process to discuss emergent themes and codes until they reached consensus and developed the final codebook. The final codebook was reviewed and finalized by the full study team.

Analyses began by double-coding six interviews (three community member, three key informant), using memos to elaborate upon the application of the codes. Differences in coding were identified and discussed until consensus was reached by the research team. The remaining transcripts were single-coded. Once all transcripts were coded, data were organized into chart format for analysis. A chart was created for each theme with summaries of different perspectives and experiences from multiple participants. This allowed the data to be compared and contrasted across different themes and perspectives [36].

This study received human subjects research approval from the Institutional Review Boards at the University of California San Francisco (#176719) and the University of North Carolina at Chapel Hill (#14–2214), as well as the Human Research Ethics Committee at the University of the Witwatersrand in South Africa (#150104). All participants provided written informed consent to participate.

## Results

We identified three specific gender norms that were influenced by the Tsima intervention and contributed to HIV service uptake: 1) men’s need to demonstrate toughness and avoidance of help-seeking, 2) men’s expected control over women in the home, and 3) women’s sole responsibility for the family’s health. Importantly, clinic-level barriers also emerged as key factors preventing HIV service uptake, particularly among men. Indeed, it is important to acknowledge the gendered nature of the health system context perceived by most participants as designed for and primarily used by women, who access HIV services as part of family planning services and antenatal care. Below, we describe how addressing specific gender norms in concert with complementary intervention strategies, supported men and women to engage in HIV services across the care cascade. We also highlight our findings related to clinic-level barriers to HIV service uptake.

### Challenging norms related to men’s toughness and avoidance of help-seeking

Participants described their perception that, prior to Tsima, many men in their community did not test for HIV because they wanted to feel and be seen as tough. To illustrate this point, several key informants noted that men did not test or go to the clinic unless they were severely ill. This was described by some to intersect with the notion that clinics were spaces for women.

[Men] used to think that they are strong, nothing happens to them…. they are saying that since they are men, they have to show their masculinity…It is rare that when a man is not feeling well [he] runs to the clinic. He just says, “I will be fine.” (Female CAT member, 24 years)

Men had this mindset that clinics are for women. Even if a man had a headache they were not doing anything about it. But since Tsima, men are going to the clinic to get tested for HIV and treatment. (Male community leader, 39 years)

A few male community members noted personally avoiding the clinic or accessing healthcare unless they were severely ill. In the following quote, one man living with HIV describes how he defaulted from HIV treatment because he did not feel sick. He describes how Tsima helped him understand the importance of continuing to take his ART even if he feels well.

As you know, I defaulted from treatment. I didn’t feel any emotional or physical pain so I talked with the (Tsima) mobilizer about why am I losing weight but I’m not sick…. Tsima helped me a lot because I was still in the dark by telling myself that at the moment I am not sick nothing bad will happen. But I learned that something bad will happen though it may take time and when it happens probably I may not be able to stand on my own. (Male community member, 24 years)

Participants also stated that many men avoided testing because they were “scared to test at the clinic.” When asked why men were scared, some participants, including community members, described that men feared they would become “stressed” if they tested positive, and believed that this stress would be psychologically unbearable and lead to a rapid decline in their health. A male community member described how Tsima helped him overcome this fear:

I was not thinking about [testing for HIV] because I was telling myself that I will be stressed if I find I’m HIV positive and I will lose weight because I know that I will die. So Tsima says you cannot die except those who want to die [by not taking HIV treatment]. (Male community member, 38 years)

The “stress” of a positive diagnosis was noted to be tied to fear of losing one’s friends and partners and general isolation due to HIV-related stigma. Two community mobilizers recounted conversations they had with men in the community about this:

One man said it is better to die not knowing his HIV status than to go and come back stressed, losing weight, plus friends will no longer drink alcohol with him. (Male mobilizer, 26 years)

[Men] are scared to share [their HIV status] with their sexual partner because [they say], “if I can tell someone that I love you but I am living with HIV that person will reject me.” (Female mobilizer, 33 years)

Mobilizers and CAT members shared their perception that men who were engaged in Tsima began to question the norm that men must always be tough. They called out intervention activities that offered an opportunity for men to come together in a space where they could confidentially and critically reflect about how gendered expectations might negatively impact their health behavior—such as avoiding HIV services—and ultimately their health and wellbeing. A mobilizer described these kinds of activities:

We have that activity “Act like a Man, Act like a Woman,” [which talks about how] a man must not cry, which is wrong. A man is a human being, he needs to cry if he feels like he wants to cry. … if he doesn’t it will keep on hurting him. (Female Mobilizer, 24 years)

In the following quote, a community member describes how participating in the intervention enabled him to question the norm that men should restrict their emotions and ultimately to access healthcare and seek support.

If I have something that hurts me inside, I don’t keep quiet I speak to my wife about it. If I am sick I go to the hospital or clinic because I don’t want to die. Many men died because they didn’t want to go to the clinic when they were sick, they keep secrets, because they are believed to be strong… If their relatives die, they do not cry… [but] you can’t be strong all the time… According to our norms, we grow up knowing that a man must be strong, so Tsima has changed that, if you feel like crying, just let your tears out. Crying heals. (Male community member, 37 years)

As illustrated in several prior quotes, informing men that someone who is living with HIV can live a long and healthy life on ART was also critical in easing fear and stress around the prospect of testing positive, in turn encouraging some men to test. Several participants also emphasized that learning how HIV testing, care and treatment could prevent onward transmission to sexual partners, thereby helping to protect their partners and children, was another motivating force for men to access HIV services. As one male community member describes in the following quote, learning that someone living with HIV can live a long and healthy life and protect their family if they start treatment early, helped ease men’s fear that they would be labeled as “sick” by others in their community and allow them to enact their gendered role of protector and provider for their family:

If it wasn’t for Tsima I don’t think I would have tested. I was scared that if I go to the clinic and get tested and find that I’m HIV positive I will be confronted with stress. But the mobilizer came and taught us the importance of knowing my HIV status and not to be scared. It does not help to know your status when you are critically ill or bed ridden. It’s good to know your status while you are walking on your feet because it will be very easy to recover on time and even people cannot notice that you are sick…So it has helped me because I managed to know my [negative] status and I have also taken my partner with me to get tested though I was scared, so today we are no longer scared. Each and every two months I make sure that I go to the clinic to recheck my status because it might happen things changed at any time…My wife can fall pregnant, if she knows that she is HIV positive our infant will be protected. (Male community member, 38 years)

### Challenging norms about male control over women in relationships

Another predominant theme that emerged from the data was related to the perception that men-controlled decision-making in the household prior to Tsima. IPV was also noted to be prevalent and was described as a common way for men to maintain control in the household and deal with disagreements. Participants described how women’s experience and/or fear of IPV prevented them from testing for HIV, disclosing a positive HIV status to their partners, and engaging in care and treatment.

I can say what is a barrier [for women getting tested] is mostly when a woman is involved in a relationship with an abusive partner…Some are scared and think that if I get tested and find that I’m HIV positive my partner will get angry with me saying that I have brought the virus in the family. (Male mobilizer, 28 years)

Participants also noted that some women who were living with HIV hid their ART because they feared their partner would find the pills, figure out they were HIV positive, and become violent. This was believed to limit women’s adherence. One mobilizer recounted the following story:

It becomes difficult [to take ART] especially if the person did not disclose their HIV status… One day we were in the support group, and someone stood up and shared her story. She is married and her husband was violent. She noticed that she was losing weight and appetite, so she went to the clinic and did HIV testing, without notifying her husband. She tested positive, and started taking treatment, but she was worried about how she could disclose to her husband. Because he was violent, she was hiding her treatment under the wood outside the house. (Female mobilizer, 39 years)

Some women also described how their partner’s coercive control prevented them from getting the care they need.

It is difficult because men do not easily accept [an HIV positive status]. I was diagnosed during antenatal care. They told me the procedures to prevent the child from being infected by HIV. Then they said I must go home and disclose my HIV status to my husband and come to start classes. I went home and told my husband that I am HIV positive…[but] I did not start taking HIV treatment because my husband…did not accept. (Female community member, 47 years)

Participants described how Tsima challenged the norm of male control over decision-making in the household through activities that facilitated critical reflection about these norms and emphasized establishing healthy relationships grounded in trust, honesty, respect, collaboration, and strong communication. In the following quote, a community member notes how challenging these gender norms helped couples in her community work together and build more collaborative relationships:

In the workshop activity, they were teaching that there are no duties for men or women. Everyone can do anything…So men have taken the lessons and tried to implement them in their families and even women have started to do the duties that were entitled for men before Tsima started in our communities. (Female community member, 27 years)

Mobilizers also noted how Tsima’s emphasis on healthy relationships and skills-building around healthy communication helped couples (who sometimes attended workshops together) and families discuss issues more openly with each other:

There is someone I know he was always fighting with his partner because there was no communication. His wife was afraid of him. If she needed something from her husband, she would not say anything because her husband was always shouting at her and abusing her physically. So, the husband attended a [Tsima] workshop and he was asking many questions. He wanted to understand some of the things [about couple communication]. The following day of the workshop he came with his wife, they attended together. After a few days, I saw them going to town and they were happy… There is a big change, they are always happy because of Tsima. (Female mobilizer, 30 years)

Participants also noted that learning about what constitutes IPV and its negative effects, as well as building conflict resolution skills to prevent it, were particularly helpful in reducing violence. For example, one community member described how he is better able to communicate with his girlfriend and not resort to violence because of his participation in Tsima:

According to what I have learned in Tsima, we must always communicate with each other. Tsima helped me, I was not communicating with her, if I wanted to do something I was doing it without communicating with her… She was always complaining about it, arguing and sometimes I was abusing her physically when she complained, but Tsima has changed that. We always communicate nowadays. (Male community member, 32 years)

Other participants noted that because of Tsima’s emphasis on trust and communication in relationships, men were better able to disclose their HIV status to their partners, and couples and families were better able to support each other to test for HIV and engage in HIV care and treatment.

For those who are attending Tsima there is a change—they are communicating and have trust in their relationships. I will give an example about a man who attended the support group… He told us that his wife did not trust him and there was no communication with her. But Tsima helped him. Now he is trusted by his wife, and they always communicate. He disclosed his status to his wife and children, and [they] remind him to take his treatment at seven o’clock in the evenings. (Female community member, 36 years)

### Challenging norms around women being solely responsible for the family’s health

As noted previously, most participants across all respondent groups said even before Tsima many women in their community were routinely testing for HIV and engaging in care and treatment. Most respondents attributed this to the health system structure, which required women to test as part of family planning services and antenatal care. Many participants also attributed women’s relatively high uptake of HIV services to their perception that women inherently value and prioritize health more than men. As one male community member (34 years) stated, “Women care so much about their health, if you can go to the clinic now, you will find many women but few men.” A female community member described the status quo as follows:

Women are testing for HIV when they go to the clinic for family planning, for antenatal clinic… When they tested for HIV [they] were also engaging in HIV treatment because women care so much about their health and the health of their children…In short, I can say women are [more] responsible for their life than men. (Female community member, 28 years)

Within this context of women’s routine use (and prioritization) of HIV and other health services, participants noted that men often inferred their HIV status based on that of their partners’, including as a way to avoid getting tested themselves. One female community member (27 years) asserted, “Previously [men] were not testing [for] HIV they were refusing to go to the clinic and relying on their woman’s status.” According to another, “…when I say let’s go for testing, he will [refuse and] say your status is my status.” (Female community member, 29 years).

Participants across all groups asserted that because of Tsima, more men were proactively testing for HIV instead of assuming their status was the same as their partner’s. They attributed this change to Tsima workshops that challenged norms that women were solely responsible for the family’s health, as well as education about sero-discordance within couples (i.e., when one partner is living with HIV and the other is not)–and particularly the importance and responsibility of both partners to routinely test for HIV. A community member described how the lessons she learned from Tsima about sero-discordance allowed her to educate her partner and convince him to test for himself.

Tsima helped me. I tested because I wanted to know my HIV status. Then I disclosed my status to my partner that I am HIV negative. He assumed that it means he is also HIV negative, but I advised him that after I attended Tsima workshops, they told us that is possible that one partner can be HIV negative while the other one can be positive. (Female community member, 24 years)

Finally, a male community mobilizer articulated Tsima’s role in encouraging men to test for HIV themselves the following way:

Mobilizers [emphasized] that everyone should go to clinic, not only women—this is not the responsibility or right for women only. It is the responsibility and right for men and women to do HIV testing. (Male mobilizer, 30 years)

### Structural barriers

Although inequitable gender norms were described as shifting because of Tsima, contributing to improved HIV service engagement for many community members, participants also noted several structural barriers that continued to prevent men from accessing HIV services. These included long wait times at health facilities (which often lasted many hours), inconvenient facility hours, and concerns that clinic staff (most of whom were women) did not maintain patient confidentiality. Participants across multiple respondent types agreed that while women also encountered and disliked these aspects of the clinic, it was men’s attendance that was most affected.

Often, the effects of these structural barriers were described in terms relating to expected gender roles for men. For instance, waiting for a long time at a facility and facility hours that conflict with work hours were perceived to be inconsistent with men’s need to work and reinforced the belief that clinics are places for women.

The first big reason for men [not attending the clinic] is queueing at the clinics, there are few men who are patient enough to queue…Besides, our clinic operates during working hours whereas men are at work between 08h00 and 16h00. Our clinics have already closed once they knock off [leave work]; they don’t have enough time to do HIV testing. (Male mobilizer, 26 years)

Women don’t mind waiting for long time at the clinic, but men are impatient on that… Because if you are a woman and you are pregnant you go to the clinic, if you want to do family planning you go to the clinic, so women are used to going to the clinic. (Female community leader, 50 years)

Additionally, although perceived lack of confidentiality at clinics was seen as an issue facing both women and men, it was particularly described as amplifying men’s anticipated stress around testing positive for HIV. This was emphasized in cases of female facility staff testing male clients.

…men at the clinic are complaining about confidentiality at the clinics especially female workers… We have few male nurses at the clinics, and [men say] females don’t have confidentiality, they talk too much. (Female mobilizer, 34 years)

…the way nurses talk to women is not the way nurses talk to men. Men felt disrespected in that way…Therefore, men don’t want to be disrespected by younger women. (Male mobilizer, 26 years)

Because the structural barriers described above were frequently raised in community consultations, intervention staff and volunteers advocated to supplement standard clinic-based testing with community-based testing events in partnership with local health care services, as part of the Tsima intervention. As indicated in the following quote, men were described to be more willing to test when the services were brought to them in the community.

[We have started facilitating] testing in their villages not at the clinic, and this has made us aware that more men are not scared to go for testing, but they are compelled by the gender norms to behave the way they do. When you check, more women are in the clinics and even the nurses are women, so men do not feel comfortable. But when you go closer to them, we see them coming out to do HIV testing in numbers. (Male mobilizer, 28 years)

## Discussion

Findings from this study in rural South Africa elucidate how a community mobilization intervention can strategically shift specific gender norms in ways that facilitate men’s and women’s engagement in HIV testing, care, and treatment. Specifically, our findings suggest that: 1) Challenging norms that men should be tough and avoid help-seeking, combined with information on the health and preventive benefits of early ART, eased some men’s fears of a positive diagnosis and facilitated uptake of HIV testing and other services. 2) Challenging norms about the need for male control over women in relationships, combined with skill-building around communication and conflict resolution, encouraged couples to consult and support each other around HIV testing and treatment. 3) Challenging the norm that women are solely responsible for the family’s health, combined with information about sero-discordance and emphasizing the responsibility of both men and women to test for HIV, encouraged men to test for HIV rather than assume their status based on their partner’s results. Focusing on addressing these three specific gender norms, complemented by improving treatment literacy and building equitable couple communication skills, could serve as an effective framework for future community-based interventions seeking to improve HIV service uptake.

Despite progress in challenging gender norms, gendered structural barriers to accessing HIV services at the facility continued to be an insurmountable barrier for many. Our findings suggest that while such clinic-level barriers to HIV service uptake were experienced by both men and women—and ultimately should be addressed for both men and women–these barriers were perceived by participants to predominantly prevent men and not women from engaging in HIV services. This finding underscores calls for addressing facility-level barriers to men’s uptake of HIV and other health services alongside community-based social and behavior change programming [37–39].

Our findings build upon prior studies in South Africa, which have also documented men’s avoidance of HIV services due to concerns of stigma and discrimination and their reluctance to access HIV services unless severely ill [1, 4, 40, 41]. As several studies have also found [1, 4], many respondents cited men’s desire to appear strong and tough as a key factor preventing them from accessing HIV services unless, or until, they were critically ill. These findings appear to conflict with results from a previous quantitative survey conducted by our own team in this rural setting, which found that while most men (55%) and women (51%) tended to endorse the statement that men should be tough, very few endorsed statements related to the importance of men’s avoidance of help seeking [3]. For example, only 27% of women and 26% of men endorsed the statement that “for men, getting sick is a sign of weakness”, and only 13% of women and 18% of men endorsed the norm that a “man shouldn’t got to a doctor unless his situation is serious” [3]. We believe this may be an example of a tendency well-documented in the field of social psychology, in which respondents’ assessment of social norms held by their peers can diverge substantially from actual norms and beliefs held by those peers (injunctive social norms) [42]. In fact, the importance of social norms around men’s avoidance of help seeking were mainly mentioned by key informants like community mobilizers, whereas when describing their own experience, male community members cited their fear of becoming “stressed” upon a positive HIV diagnosis as a main reason for their avoidance or delay of HIV testing. Such fear and stress has also emerged in other research among men in South Africa; as in our study, this fear and stress has been seen as potentially debilitating and related to the drastic change an HIV positive diagnosis can have on their identity and how they live their lives [43, 44].

The anticipated stress of a positive diagnosis may also be why treatment literacy emerged as an important factor in alleviating fears and stress around restrictive gender norms and HIV stigma. Understanding ART and onward prevention helped change the belief that a positive diagnosis would entail fundamental changes to men’s lives, including their ability to live up to perceived expectations of themselves as men. Learning that early HIV treatment would enable one to stay healthy, continue to provide for one’s family and maintain independence, was described to motivate participants to test and access treatment. Some respondents also described that the knowledge that treatment prevents onward transmission, which was new to most, further incentivized men to find out their status and access treatment to protect their partners and children. This corroborates previous work by our team, which also found that men were motivated to engage in HIV services to prevent onward transmission [45]. However, our research conducted with service providers in this setting around the same time as the present study, found that only 42% of HIV service providers were informed about treatment as prevention, and among those, only 61% said they always shared this information with their HIV-positive patients [46]. Considering this, it is critical to improve provider communication with their patients about treatment as prevention, and to improve community education about this topic.

Our findings also suggest that Tsima resulted in a notable shift towards more equitable decision making and improved communication among couples involved in the intervention, including open discussion and support for HIV testing and treatment. Further, our qualitative findings support the results from our quantitative research which documented a significant reduction in reported IPV among women enrolled in the Tsima intervention arm, as compared to the control [47]. Tsima activities that provided communication and conflict resolution skills-building seemed to play an important role in facilitating these changes. Prior research, including at the study site, has also recognized the role of couple communication and conflict resolution in facilitating HIV prevention, testing and treatment [3, 48, 49], as well as reducing IPV [47, 50, 51]. For example, previous interventions have had success in increasing HIV testing by working with couples to improve their communication skills and problem solve around barriers to talking together about HIV risk [48, 49]. We also found that increasing knowledge about sero-discordance, and framing testing as a responsibility and right for both members of the couple, encouraged more men to start testing for themselves and reduced the onus of HIV testing on women.

Finally, findings from this study highlight features of health facilities that reinforce and institutionalize the gender divide in norms and practices around accessing HIV services. This was exemplified in participants’ testimonies that most women have regular access to HIV testing, care, and treatment due to the integration of these services into family planning and antenatal services, while men do not–as other research has also found [4, 52, 53]. This disparity in access to services was described to fuel the perception that the health system was designed for women and reinforced the gendered belief that women prioritize health more than men. Further, while both men and women were described to face the same barriers to service uptake, participants uniformly asserted that men were deterred by such obstacles, whereas women were not. Although participants often attributed this difference to women inherently valuing and prioritizing their health more than men, we posit that this difference is driven by the health system structure and the lingering belief that only women (as opposed to both partners in a couple) are responsible for family planning, antenatal care, and childhood wellness. As suggested by some of the participants in this study, until health facilities can better meet men’s needs, and are perceived by men to do so, HIV services may need to be offered in community settings as well. Indeed, when Tsima partnered with public clinics to do so, many men readily took up these services. Recent research has also found HIV self-testing (HIVST) to be a promising and acceptable approach to HIV service delivery among men and other groups who feel less comfortable accessing health facilities [54–56]. With roll out of HIV self-testing gaining momentum in South Africa [57], HIVST may improve testing uptake among men in this setting. However, our findings suggest that this approach must be paired with acceptable strategies to improve linkage to and retention in HIV care and treatment among this population.

Regarding limitations of the study, it is important to consider that these findings reflect the unique context of rural South Africa and may not apply to other contexts. Also, the purposive sampling of intervention participants and staff may limit generalizability of findings to the community more broadly. In fact, in analyses that explored changes in gender norms using household survey data from the 2014 baseline to the 2018 endline for the Tsima trial, we found that there was a strong secular trend towards more equitable gender norms in both intervention and control villages [47]. In addition, it is possible that participants may not have felt comfortable providing critical feedback related to the intervention or openly discussing the socially charged topic of gender norms and HIV service uptake with the interviewers. To mitigate this, we utilized highly experienced interviewers trained in qualitative methods, including neutral probing and strategies to establish rapport, and who were not part of the intervention team. Nevertheless, social desirability bias remains a potential concern for sensitive topics, although the similarity in participant perspectives and frequency of agreement underscores the strengths of our findings.

## Conclusion

Findings from this study shed light on how community mobilization efforts can shift key inequitable gender norms linked to HIV service uptake, and positively effect engagement in HIV services. Among these key gender norms found in the context of rural South Africa were norms around men’s toughness/avoidance of help seeking, men’s power and control in relationships, and women’s role as primary caregiver for the family. While these norms are common in many settings, it is important to reaffirm and distinguish specific relevant gender norms in a given setting when tailoring related programs. Our findings also highlight the promise of the Tsima strategy of pairing intervention messages and activities that challenge these norms with those that provide concrete and up-to-date information related to HIV care and treatment and skills around equitable couple communication and conflict resolution. Health facility-related structural barriers continued to prevent some men from engaging in HIV service, despite community mobilization efforts, suggesting that the strategies outlined above should be coupled with approaches that address facility-level barriers to service uptake [37–39].
